# Priming of Frames and Slots in Bilingual Children’s Code-Mixing: A Usage-Based Approach

**DOI:** 10.3389/fpsyg.2021.726764

**Published:** 2021-10-22

**Authors:** Antje Endesfelder Quick, Dorota Gaskins, Maria Frick

**Affiliations:** ^1^Faculty of Philology, Institute of British Studies, University of Leipzig, Leipzig, Germany; ^2^School of Education, Communication and Society, King’s College London, University of London, London, United Kingdom; ^3^Department of Languages and Literature, Faculty of Humanities, University of Oulu, Oulu, Finland

**Keywords:** code-mixing, usage-based, lexically specific patterns, priming, corpus linguistics

## Abstract

This article investigates the role of direct input in the code-mixing of three bilingual children aged 2–4 years acquiring English as one language, and either German, Polish, or Finnish as the other. From a usage-based perspective, it is assumed that early children’s utterances are item-based and that they contain many lexically fixed patterns. To account for such patterns, the traceback method has been developed to test the hypothesis that children’s utterances are constructed on the basis of a limited inventory of chunks and frame-and-slot patterns. We apply this method to the code-mixed utterances, suggesting that much of the code-mixing occurs within frame-and-slot patterns, such as *Was ist X?* as in *Was ist breakfast muesli?* “What is breakfast muesli?” We further analyzed each code-mixed utterance in terms of priming. Our findings suggest that much of the early code-mixing is based on concrete lexically fixed patterns which are subject to input occurring in immediately prior speech, either the child’s own or that of her caregivers.

## Introduction

The predominant goal of language acquisition studies is to understand how children acquire language(s) and why they produce certain structures the way they do. Bilingual children are of particular interest to those studying input-output relations: not only is their input spread across more than one language but, most notably, languages in contact almost certainly influence each other.

Code-mixed utterances, which use two languages in one utterance (e.g., *Das ist the next job* “This is the next job”), are a salient outcome of language contact. For decades, researchers have analyzed code-mixed utterances in adults as well as children and produced a wealth of possible explanations. Strictly linguistic approaches to mixing are probably best known for their search for universal syntactic constraints on the phenomenon (see Gardner-Chloros, 2009 for an overview) as they intend to explain where in an utterance a switch from one language to another may occur, and which elements may be switched (generally the distinction is between lexical and grammatical elements). However, focusing mainly on syntax and form, these approaches neglect the role that cognition plays in the selection of words, patterns, and other linguistic elements. An important aspect of cognition is entrenchment (e.g., Schmid, 2020), understood as memory consolidation and central to how patterns are stored and retrieved. Frequently occurring patterns become entrenched in memory and are more easily activated and produced. This is exactly where the usage-based (UB) approach steps in.

The UB framework has only recently started to be adopted to work on multilingualism (see Backus, 2021 for an overview). Decades of UB research on monolingual acquisition had highlighted that children’s acquisition proceeds in a piecemeal fashion starting with whole words and chunks (holophrases) and a limited number of recurrent patterns, before abstracting away increasingly complex patterns from their linguistic experience (see e.g., Lieven et al., 2003, 2009; Tomasello, 2003; Ambridge and Lieven, 2011). Children’s growing productivity therefore is based on the reuse of already acquired formulaic language (chunks/patterns/frames). If we extend this idea of recycling and reusing already acquired and entrenched patterns to bilingual contexts, code-mixed utterances such as *Das ist the next job* “This is the next job” can be analyzed in terms of combining constructional patterns from two languages. These patterns have been already acquired and have been used in the children’s language and, consequently, we can assume that they are deeply entrenched. These are called frame-and-slot patterns (*Das ist* X), with a frame placed utterance initially into which a slot filler (X) is inserted (e.g., Quick et al., 2021).

An assumption characteristic of the UB framework is that there is an intimate relationship between the linguistic knowledge and the instances of language use: input provides the basis from which children extract and build up their linguistic knowledge. Immediate discourse effects have been shown to play a major role in the production of constructions, particularly in the form of priming (e.g., Kirjavainen and Theakston, 2011; Koch et al., 2020). In this article, we wish to investigate if and how the immediate discourse influences children’s production of code-mixed utterances in German-English, Polish-English, and Finnish-English. We first analyze all code-mixed utterances in terms of pattern use adopting a UB perspective and, secondly, in light of their precedents in the immediate discourse.

## Language Acquisition From a Usage-Based Perspective

Language use is one of the central criteria that define humans and therefore, it is not surprising that many researchers have tried to uncover the mechanisms underlying acquisitional processes (e.g., Ambridge and Lieven, 2011). The predominant goal of language acquisition studies is to understand how children learn language. For a long time, research in child language has been dominated by nativist assumptions, arguing that language input is so impoverished that children’s rich knowledge of grammar could not possibly be extracted from it (Chomsky, 1965). To avoid the problem of impoverished input, proponents of the nativist perspective suggested instead that children are equipped with an innate universal grammar and all languages share one set of underlying syntactic principles of grammar.

Over the years, an increasing number of researchers have challenged the concept of the “poverty of stimulus” and claimed that children are able to extract linguistic knowledge from the input through learning mechanisms which are responsible for most other types of learning (see Ambridge and Lieven, 2011 for an overview, Tomasello, 2003). The UB approach relies on the assumption that humans build up knowledge (or “mental representation,” or “competence”) on the basis of their experience. As such, UB approaches counter the generative position of a universal grammar and view linguistic knowledge as emerging from item-specific learning. Linguistic constructions (form-function pairings) are the basic building blocks of grammar. What children acquire is a growing inventory of constructions that move along a continuum of varying complexity and different levels of abstractness, ranging from lexically specific units, or “chunks” (*What’s up*), via partially schematic or frame-and-slot patterns (*I want X*), to fully schematic schemas (*Pronoun VP NP*). Frame-and-slot patterns are a vital link on a continuum of schematicity. Children learn multi-word units which subsequently become analyzed as frame-and-slot patterns, creating open slots. By definition, this means that the construction becomes productive; the growing number of its different instantiations may finally give rise to the ultimate schematization: the abstract grammatical pattern.

From a methodological point of view, the question arises how to account for patterns in children’s speech. To identify chunks and frame-and-slot patterns, the *traceback method* has proven to be a very reliable operationalization (e.g., Lieven et al., 2009, for an overview see Hartmann et al., 2021). The basic idea of the traceback method is to account for a set of so-called target utterances by tracing them back to the previous utterances. In this way, the method identifies recurrent patterns that are assumed to be cognitively entrenched. To do this, a corpus is usually split into two parts, called the main corpus and the test corpus. The test corpus contains those utterances (usually the last recording in a corpus) which are “traced back” to the earlier utterances which constitute the main corpus. The underlying rationale of this method is to identify recurring chunks/patterns and frame-and-slot patterns in the test corpus. These patterns are subsequently traced back to earlier utterances which constitute the main corpus. If utterances occur verbatim in previous recordings, a chunk is established, e.g., *I want it*. If the target utterance is matched only partially, the procedure can yield a frame-and-slot pattern such as (*I want* X). The method starts from the assumption that children’s linguistic output can be attributed to a limited number of patterns which are reused with different slot fillers and thus contribute to the increasing productivity and creativity. Consequently, this method is a purely data driven, bottom-up approach which only identifies patterns if the children uttered them before, reflecting the piecemeal acquisition by relying on general cognitive learning mechanisms such as pattern finding, imitation, and the entrenchment of frequently encountered patterns (Tomasello, 2003; Schmid, 2020). In various studies based on the traceback method, the patterns that can be identified in children’s early speech have been shown to depend to a large extent on individual input (see, for example, Ambridge and Lieven, 2011; Quick et al., 2019). The traceback method is yet to be applied to the study of input effects on bilingual children’s early linguistic resources (but see section “Code-Mixing in Bilingual Children” for an explanation of how it was applied to the study of code-mixing).

### The Role of Input

In UB approaches to language acquisition, input plays a major role since the linguistic knowledge children accumulate throughout their development is developed directly from the input: language therefore, is learnable with the help of basic cognitive and social-cognitive processes (e.g., imitation, analogy, automatization, and entrenchment) (e.g., Cameron-Faulkner et al., 2003; Kirjavainen et al., 2009; Quick et al., 2018). Input differs considerably across children. What we find normal for adult speakers also holds, up to a point, for young children: they have different language experiences concerning the number of languages, the topics of conversation, the types of interlocutors and conversational settings, and this implies differences in the quality and quantity of their linguistic experience. As such, input and output are inextricably linked to each other and research on child-directed-speech (CDS) has shown that input is very repetitive and that caregivers often use recurrent utterance initial frames which are subsequently picked up by the children (Cameron-Faulkner et al., 2003; Stoll et al., 2009). Cameron-Faulkner et al. (2003) analyzed the speech of English-speaking caregivers addressed at children and showed that children’s language productions often contained reproductions as well as manipulations of linguistic constructions experienced in caregiver speech. Caregivers’ speech often contained frame-and-slot patterns such as *Let’s X*, which were subsequently picked up by the children. These findings suggest that children learn multiword units directly from the input. Since English has a relatively fixed word order, Stoll et al. (2009) conducted a similar study comparing the CDS in Russian and German-speaking mothers, two languages with a more flexible word order. The results obtained were similar to the English CDS analysis: mothers were very repetitive in their use of utterance initial sentence frames.

It remains to be seen whether these results also hold for bilingual children. There is, however, substantial evidence that acquisition in early bilingualism is driven both by the quantity and quality of linguistic experience in children’s two languages (for a review see Unsworth, 2016). For example, the rates of vocabulary learning in the second year of life closely reflect the amount of input children receive in each of their two languages (Pearson et al., 1997; David and Li, 2008; Gaskins, 2020). To an extent, input is also responsible for morphosyntactic acquisition. Hoff et al. (2012) report that children aged 22–30 months and dominant in one language tend to produce grammar consistent with their peers but in their less dominant language they lag behind their monolingual peers in terms of the presence of word combinations, grammatical complexity and the mean length of the three longest utterances. However, Barreňa et al. (2008) report that as little as 60% exposure is reliable in predicting grammatical development within monolingual norms up to the age of 30 months.

Apart from the general assumption that children can learn language from their input, direct discourse situations also have an immediate effect on children’s productions. Kirjavainen et al. (2009), for example, showed that some errors children make, e.g., me-for-I errors, can be explained by priming. If *me* occurs in the direct input before non-finite verbs (e.g., *Let me do it*), it gives rise to the occurrence of “me do” errors in the children’s speech. In another study, Kirjavainen and Theakston (2011) showed that children’s production of *Want to VP* and *Want X* constructions was influenced by the occurrence or absence of the respective construction in the immediate discourse.

Since immediate discourse has a direct influence on production, we want to extend this idea to code-mixing to investigate if discourse effects also play a role in the production of code-mixed utterances.

### Code-Mixing in Bilingual Children

Code-mixing (CM) is most broadly defined as “*the alternative use by bilinguals of two or more languages in the same conversation*” (Milroy and Muysken, 1995, p. 7), including mixing within the same utterance (e.g., *Wo ist paper* “Where is paper”). It is a salient phenomenon that can be observed in language contact situations, including bilingual first language acquisition and as such, has been the subject of considerable research effort. Initial CM research was largely focused on sociolinguistic and pragmatic domains following research questions such as why people engage in multilingual language use. Linguistically oriented studies, however, often tackled the issue of what the underlying structures and rules are that govern CM (an early landmark was Poplack, 1980; see Gardner-Chloros, 2009 for an overview). In the linguistic tradition, a large body of research was produced investigating the issue of universal syntactic constraints, attempting to find out where and when in a sentence a switch is permissible and where not (i.a. Poplack, 1980; Di Sciullo et al., 1986; Belazi et al., 1994; Myers-Scotton, 1997; MacSwan, 2000). However, none of the suggested constraints have been proven robust enough to withstand attempts to find counterexamples (see Quick and Verschik, 2019 for an overview). While focusing on form alone, linguistic accounts neglected to look at meaning and how we conceptualize it.

Recently, CM has been taken onto new grounds explaining CM through reference to usage, particularly to chunking and entrenchment processes (e.g., Gaskins et al., 2019; Backus, 2021; Quick et al., 2021). From a UB perspective, CM can be understood as a process of pattern activation from two languages. For example, *Komm her police sheep* “Come here police sheep” can be analyzed as a frame-and-slot pattern *Komm her X* into which the lexical item *police sheep* is inserted. But open slots can be filled with more than just lexical items, as in the case of *Wir müssen this löschen* “We have to extinguish this” which contains a frame *Wir müssen X* “We have to X” and an open slot *this löschen* “extinguish this.” In such studies, the aforementioned traceback method has also been applied to the investigation of code-mixed utterances in bilingual children showing that bilingual utterances are also constructed around frame-and-slot patterns which had been used before and show a high degree of formulaic nature (e.g., Quick et al., 2018). The authors have deviated from the convention to use the last sessions of recording as test corpus and the remaining dataset as main corpus. Instead, Quick et al. (2018) used all code-mixed utterances as test corpus and traced them back to the earlier utterances which constituted the main corpus. Quick et al. (2018) found that a German-English bilingual child’s code-mixing contained many frame-and-slot patterns such as *This is X* as in *This is kein taxi* “This is no cab” but also completely lexically fixed bilingual patterns (chunks), such as *Und this* “And this” which the child constantly reused in this form. Not only did monolingual acquisition studies show that children’s early utterances can be accounted for by a limited number of recurring patterns and frames, but mixed utterances can also demonstrate the key relevance of lexically fixed patterns and frame-and-slot patterns. The authors further showed that many of the attested patterns could be traced back to the direct input providing evidence that children can extract linguistic knowledge from the input (Quick et al., 2018, 2019). Thus, lexically fixed patterns and/or open slots were influenced by the immediate discourse, especially at a younger age when the children were less proficient in their language(s) and the degrees of entrenchment were relatively low. Discourse effects (priming) may thus serve as a lifeline for children who are eager to communicate but whose linguistic knowledge is not sufficiently entrenched to offer words and combinations which could be retrieved in a given communicative situation. On the basis of the above, we wish to analyze the CM of three bilingual children growing up with different languages (English-German, English-Polish, and English-Finnish) and to test if (a) CM mostly occurs within frame-and-slot patterns and (b) if parts of the mixed utterances are inferred from the immediate discourse. A UB approach is particularly well suited to this type of analysis since it puts constructional patterns as well as input-output relations center stage.

## Methods

### Participants

Three bilingual children were included in the study. All data were recorded at home in the children’s familiar environment. All children had English as one of their languages and either German, Polish, or Finnish as their other language. Whereas Fion and Sadie grew up as simultaneous bilinguals, Eetu is a successive bilingual.

The first child is Fion, who grew up in Germany with a German-speaking mother and an English-speaking father. Fion was the second child in the family and had an older brother who was also raised as a bilingual. Both parents adhered to the *One parent-one language* (OPOL) strategy when speaking to the child. Fion’s data covered a span from 2.3 to 3.11, however, for the present analysis we only used the recordings at the ages of 2.3, 3.0, and 3.11 (total *n* = 5443, mixed *n* = 321). The corpus is a highly dense corpus which means recordings took place 3–5 times per week for 1 h. The recordings took place in a bilingual context with both parents being present. Hence, it was not possible to ascertain what type of context was most conducive to CM. Since Fion lived in Germany and went to a German kindergarten his input is mainly German. Fion’s acquisition of slot and frame schemas is further discussed elsewhere in literature, for example in relation to how his CM patterns reflect the conditions of his changing input (Quick et al., 2018).

The second child included in our study is Sadie, a Polish-English bilingual child. Sadie’s mother was a native speaker of Polish and her father a native speaker of English. The family lived in England and thus, Sadie was exposed to more English than Polish. Sadie’s data covered a span from 2 to 2.5 years of age (total *n* = 1,734, mixed *n* = 244). In total, there are 30 recordings available: 10 with Sadie being addressed in Polish by her mother, 10 with Sadie being addressed in English by her father, and 10 with the child being spoken to in Polish by her mother, and English by her father. Sadie produced the most mixed utterances when addressed in Polish (*n* = 142), and the least when addressed in English (*n* = 20). Sadie’s acquisition of slot and frame schemas is also discussed elsewhere in literature, for example showing links between the segmentation of constructions and switch placement (Gaskins et al., 2019).

The third child is Eetu, who initially grew up with Finnish only, but from the age of 2.2 an English-speaking live-in au-pair looked after him throughout the day. She addressed Eetu in English for the most part and thus, he was exposed to English most of the day. Eetu is dominant in Finnish and except for the au-pair, had no other source of English. The recordings took place in a bilingual context at rather infrequent intervals. This made it impossible to determine what type of context might trigger the most code-mixing. The data used in this study covered a span from 2.9 to 2.11 (total *n* = 516, mixed *n* = 137).

### Data Analyses

In a first step, all utterances were coded for language type: monolingual utterances and CMed utterances. Secondly, for each of these utterances we analyzed the mean length of utterances (MLU) so that we were able to compare the MLUs of the monolingual as well as CMed utterances.

In our main analysis, we followed Gaskins et al. (2019) and analyzed all mixed utterances according to their level of schematicity. Mixed utterances could fall into four categories:

(a) Completely lexically fixed which renders a chunk, e.g., *Daj me that* “Give me that” (from Sadie).
(b) A combination of two chunks, e.g., *Das ist* + *the next job* as in *Das ist the next job* “This is the next job” (from Fion).
(c) A frame-and-slot pattern, e.g., *And this is X* as in *And this is tässä* “And this is here” (from Eetu).
(d) Other, e.g., *Echt cars* “Real cars” (from Fion).

In order to account for chunks and partially schematic units, we used the *traceback method* which traces utterances or parts of utterances back to previous utterances in the corpus. A chunk is only identified as a chunk if it can be related back verbatim to at least one previous utterance in the recordings. If the traceback only yields a partial match, a partially schematic utterance is instantiated (example c). Utterances which contain a creative combination of two chunks are coded as chunk + chunk (example b). All utterances which do not contain a recurring part are coded as other (example d).

In our second analysis, we were interested in whether parts of the code-mixed utterance are influenced by the discourse (priming). To determine this, we examined 20 utterances prior to the child’s turn (including child and caregiver utterances) (Table 1). If priming did occur, we first checked which part of the code-mixed utterances had been primed (i.e., the lexically fixed part, the frame in a frame-and-slot pattern or the open slot in a frame-and-slot pattern). As a second step, we identified the source of the prime. The child could either prime him/herself (self-priming) or the interlocutor could be the source of priming (discourse priming). In Table 1, there is an example of CM from Fion’s data (*Was ist breakfast muesli*?) which was partly primed, in this case by the father. Fion’s CM consists of a frame *Was ist* X “What is X” and an open slot X filled with *breakfast muesli*. In this specific example, the open slot X was primed by the father *breakfast muesli* which Fion picked up in his code-mixed utterance *Was ist breakfast muesli*? “What is breakfast muesli?”

**TABLE 1:** Example discourse priming.

**Fion, age 3**	**Frame-and-slot**	**Open slot X**	**Discourse situation**
FAT: We have breakfast muesli	We have X	Breakfast muesli	Input
CHI: Was ist breakfast muesli?	Was ist X	Breakfast muesli	Output
			

## Results

### Mean Length of Utterances and Frame-and-Slot Patterns in Code-Mixing

Our first analysis was concerned with the length of the different utterance types (Table 2). The MLUs were calculated in words separately for the monolingual and CMed utterances. In general we found that each child’s input situation was reflected in his/her MLU scores. The language with the greater input contributed to longer utterances in that language. Fion’s input was predominantly German which is also reflected in a longer MLU for the German utterances compared to the English utterances. Sadie’s predominantly English input was also mirrored in her MLUs, with English utterances being longer than her Polish utterances. Eetu, our successive bilingual, had a similar MLU in Finnish and English.

**TABLE 2:** Mean length of utterances (MLU) for the monolingual and code-mixed utterances.

**Fion**	**German**	**English**	**Code-mixed**
2.3–3.11	2.9	2.1	4.1

**Sadie**	**Polish**	**English**	**Code-mixed**

2–2.5	1.3	2	2.8

**Eetu**	**Finnish**	**English**	**Code-mixed**

2.9–2.11	1.9	1.8	3

We also analyzed the MLU for the code-mixed utterances and this showed an interesting picture. For each child we observed that the code-mixed utterances were longer than the monolingual utterances, even in comparison to each child’s more proficient language.

**FIGURE 1:**
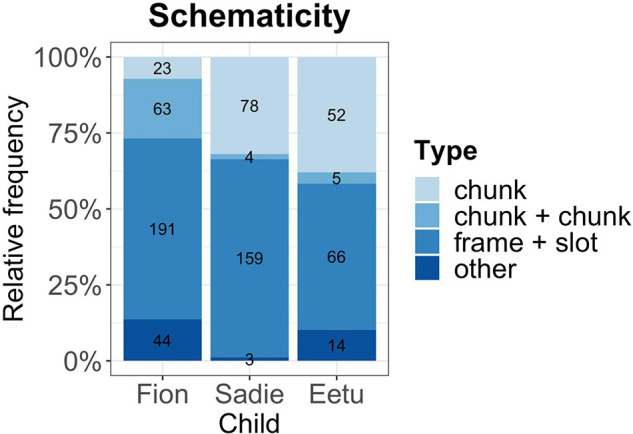
Schematicity in code-mixing. Numbers in the bars indicate the number of cases.

Our main analyses was concerned with the building blocks in the mixed utterances. As detailed above, we looked for recurring units, such as chunks and frame-and-slot patterns in CM (Figure 1). Results were supported by a Chi^2^ test and showed that most of the time children’s CM is constructed around lexically fixed patterns, either as a complete chunk or as a partially schematic utterance [*X*^2^ (6, *N* = 697) = 140.0731, *p* = 0.001]. For example, Eetu used a frame in the mixed frame-and-slot pattern: *It is X* as in *It is sinistä kakkua* “It is blue cake.” Likewise, Fion produced a bilingual utterance *Die sind for daddy* “These are for daddy” which contains two chunks, *Die sind* and *for daddy.* We also found instances of chunks which are bilingual, such as Sadie’s English-Polish chunk at the age of 2.2 *And to* “And this.” However, it also has to be noted that the children show individual differences in their use of chunks and patterns. For example, Eetu and Sadie used more chunks in their CM which could be due to their dominance in one of their languages (Finnish for Eetu, and English for Sadie), or the amounts of data we have for each child. Looking at the language (monolingual or mixed) of the chunks/frames, we see that in Fion’s case 50% of the frames were realized in German, whereas Sadie and Eetu used overwhelmingly English in their frames (83 and 66%). Despite their different profiles and dominance levels, all children rely considerably on lexically fixed items in their mixed utterances (for a complete analysis of the data set see Gaskins et al., 2019).

### Discourse Effects on Code-Mixing – Parental and Self-Priming

**FIGURE 2:**
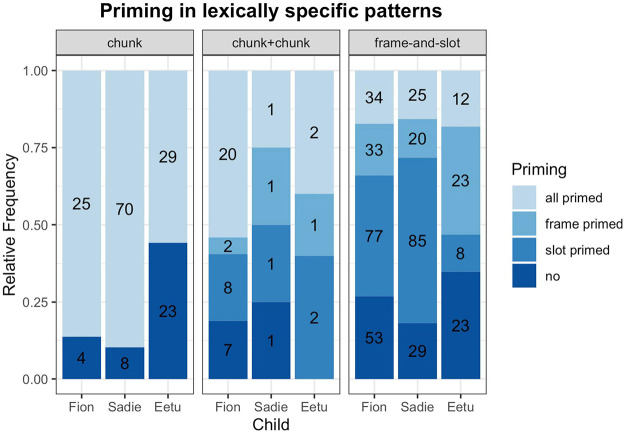
Priming in lexically specific patterns. Numbers in the bars indicate the number of cases.

In our second analysis, we were interested in how far prior speech influences the production of the mixed utterance. We used all bilingual utterances from our first analyses for which we could identify a chunk or frame-and-slot pattern (*n* = 641) and examined if the complete code-mixed utterance, or parts of the utterance, had occurred in the previous 20 utterances (see Figure 2). Under three possible scenarios, either the whole chunk, or a frame, or an open slot in a frame-and-slot pattern could be primed. In Figure 2 we can see that priming plays an important role, irrespective of the primed parts. Children’s CM often consisted of parts which had been provided by the direct discourse [*X*^2^ (6, *N* = 641) = 35.4042, *p* = 0.05].

**FIGURE 3:**
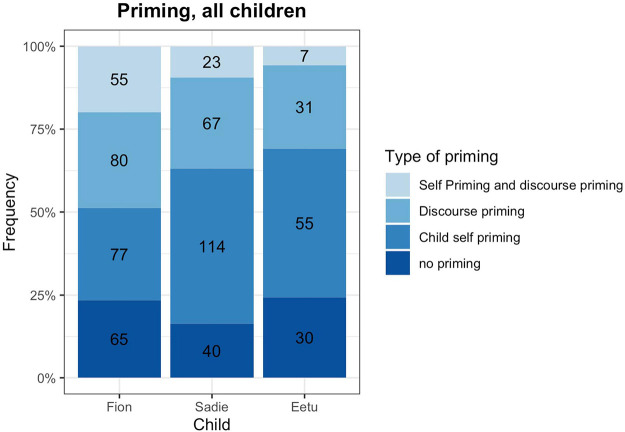
Type of priming. Numbers in the bars indicate the number of cases.

Finally, we also analyzed the source of priming. As such, priming could either happen through the child herself in the form of self-priming, the discourse with the interlocutors, or both child and discourse could provide the prime. First of all, Figure 3 shows that priming is pervasive and that for most code-mixed utterances we can find precedents in the immediate discourse, either provided by the child him/herself or by the interlocutors. If priming did occur, very often children were their own source of the prime, especially when they produced bilingual chunks. But also the discourse provided a substantial number of priming instances which children picked up to construct their bilingual utterances [*X*^2^ (4, *N* = 509) = 30.91, *p* = 0.001]. Interestingly, even for complete bilingual chunks we see that children pick up their own realizations and prime themselves. For example, Fion continuously used the bilingual chunk *Und this* “And this” which he had not encountered in the discourse but which had become temporarily entrenched through his own practice. For chunk + chunk combinations, we see a slightly different picture. Whereas Fion’s chunk + chunk combinations were often primed completely, Sadie and Eetu did not show a specific pattern (although we have to be careful with conclusions due to the low numbers of such combinations). Another interesting picture emerges for the frame-and-slot pattern priming: Fion and Sadie mostly show slot priming but most of Eetu’s frame-and-slot patterns are either not primed, or the first part, the frame, is primed. This shows that also the immediate discourse situation influences the construction of code-mixed utterances.

## Discussion

In this study, we analyzed CM from a UB perspective in three bilingual children growing up with different language combinations. In our main analysis, we concentrated on the role which lexically fixed patterns play in CM. Results showed that all three children constructed their mixed utterances around frame-and-slot utterances, such as *And me go X* as in *And me go pois* “And me go away” (Eetu). However, with the group case study approach adopted in this article, comparisons between the proportions of different construction types are difficult as the children display different language profiles, and we have very different amounts of data for each child. Secondly, we investigated the role of the discourse in the form of priming, showing that very often parts of the mix had been provided by the direct discourse. Thirdly, we investigated the source of the priming, and found that children were primed both by themselves and by their interlocutors.

From a UB perspective, it is assumed that children acquire their languages in a piecemeal fashion by relying on lexically fixed and partially schematic patterns. In order to do so, children use general cognitive learning mechanisms, such as pattern finding or the entrenchment of frequently encountered units (Tomasello, 2003; Schmid, 2017). The same process can be applied to mixed utterances. Instead of analyzing where and when in an utterance a switch is possible or which items (lexical vs. grammatical) can be inserted, mixed utterances can also be analyzed in terms of patterns and entrenchment processes (e.g., Quick et al., 2021). Whatever is fixed is easier to store and consequently easier to activate (Schmid, 2017). Frequently occurring and used forms become better entrenched and consequently easier to activate without close monitoring. Thus, well entrenched patterns also find their way into code-mixed utterances. From this perspective, CM can be analyzed as an activation phenomenon: some words, chunks and/or frame-and-slot patterns are activated faster in one language because they are better entrenched and our MLU results support this idea. Codemixed utterances are consistently longer than the monolingual utterances. If CM would be merely a lexical gap-filling strategy MLUs would not differ between the mono- and bilingual utterances. It seems like children can exploit their two languages instead of just one language and consequently are more communicative.

Concerning the identification of patterns, the traceback method is a purely data-driven method which detects patterns only if they occurred before. As such, it is a rather conservative method which probably underestimates the number of occurrences as the corpora used in this study only capture a small percentage of the children’s daily speech.

Since input and output are inextricably linked to each other, UB accounts assume that children can extract linguistic knowledge from their immediate environment. Therefore, our second analysis was concerned with how much material can be provided for children to use in their CM through prior speech. Our analysis of priming indicates that in the case of each child prior speech plays an important role in the construction of code-mixed utterances. Children often use structures which occur immediately prior to their own turns. In our data, we found that children were primed both by themselves and by their interlocutors.

In general, priming lowers the processing burden since patterns and words had been activated before and thus, they are easier to retrieve (e.g., Schmid, 2017). Strongly entrenched patterns and words can be retrieved more easily from long-term memory, but especially less entrenched patterns can be activated by priming in the immediate discourse and in turn also become more entrenched in this process; Schmid refers to this as a feedback loop (Schmid, 2017, 2020). This is a particularly important strategy for monolingual children who are two (less so 3 years of age) as for these children constructions are not yet fully entrenched (Koch et al., 2020). Our study provides evidence that priming also applies to bilingual acquisition between their second and third birthday.

In all three children studied, output therefore closely follows the input situation, an observation which echoes previous usage-based findings (e.g., Kirjavainen and Theakston, 2011). Priming is a pervasive and immediate input effect which plays an important role especially in language acquisition and even in the production of highly creative utterances such as CM. Even though mixed utterances appear to be creative, the bits and pieces that are used in them are not, as they are taken either from the child’s existing repertoire of entrenched patterns, or from caregiver input.

Our study contributes to the understanding of how code-mixed utterances are assembled. In the vast majority of cases, they are put together partly from the elements that the child can access and retrieve from memory, and partly from elements which are reinforced by the immediate discourse. In all cases such elements are independent chunks which are “patched up” together to meet these very young children’s communicative needs.

Lastly, while our group case study approach allowed us to capture similarities in the use of CM, any observations made here should be treated with caution. In order to verify how our results hold in larger populations of children, the traceback method needs to be applied more widely, ideally in its automated form to allow simultaneous analyses of several large datasets (Quick et al., 2019), and even subjected to experimental design (Bannard and Matthews, 2008).

## Data Availability Statement

The raw data supporting the conclusions of this article will be made available by the authors, without undue reservation.

## Ethics Statement

Ethical review and approval was not required for the study on human participants in accordance with the Local Legislation and Institutional Requirements. Written informed consent to participate in this study was provided by the participants’ legal guardian/next of kin.

## Author Contributions

All authors listed have made a substantial, direct and intellectual contribution to the work, and approved it for publication.

## Conflict of Interest

The authors declare that the research was conducted in the absence of any commercial or financial relationships that could be construed as a potential conflict of interest.

## Publisher’s Note

All claims expressed in this article are solely those of the authors and do not necessarily represent those of their affiliated organizations, or those of the publisher, the editors and the reviewers. Any product that may be evaluated in this article, or claim that may be made by its manufacturer, is not guaranteed or endorsed by the publisher.
